# TDB protects vascular endothelial cells against oxygen-glucose deprivation/reperfusion-induced injury by targeting miR-34a to increase Bcl-2 expression

**DOI:** 10.1038/srep37959

**Published:** 2016-11-25

**Authors:** Li-Xi Liao, Ming-Bo Zhao, Xin Dong, Yong Jiang, Ke-Wu Zeng, Peng-Fei Tu

**Affiliations:** 1 State Key Laboratory of Natural and Biomimetic Drugs, School of Pharmaceutical Sciences, Peking University, Beijing 100191, China

## Abstract

Prolonged ischemia can result in apoptotic death of vascular endothelial cells and lead to ischemic vascular diseases including vascular dementia, arteriosclerosis and brain oedema. Finding protective strategies to prevent this is therefore an urgent mission. Recent studies have shown that dysregulation of microRNAs (miRNAs) can lead to imbalance of Bcl-2 family proteins and mitochondrial dysfunction, leading to further damage of vascular cells under ischemic conditions. However, whether miRNAs can be used as a drug target for treating vascular diseases is not fully understood. In this study, we observed that the natural product 2,4,5-trihydroxybenzaldehyde (TDB) could effectively inhibit vascular cell apoptosis following oxygen-glucose deprivation/reperfusion (OGD/R) by maintaining mitochondrial membrane potential (MMP) and suppressing activation of the mitochondria-dependent caspase-9/3 apoptosis pathway. Furthermore, we identified miR-34a, a crucial negative regulator of Bcl-2, as a target for the protective effect of TDB on vascular cells. TDB-induced suppression of miR-34a resulted in a significant upregulation of Bcl-2 protein, MMP maintenance, and the survival of vascular cells following OGD/R. Our findings suggest that targeting miR-34a with the natural product TDB may provide a novel strategy for the treatment of ischemic vascular injuries, and demonstrate the therapeutic potential in targeting miRNAs using appropriate small molecules.

Vascular endothelial cells are one of the primary targets of ischemic vascular injury, and their damage has been repeatedly shown to cause various vascular dysfunctions. Although several studies have suggested the importance of protective strategies for vascular endothelial cells^1^,^2^, there are still no effective strategies and agents for treating vascular damage against ischemia insult. Mitochondria, the most important energy-producing organelles in cells, regulate numerous physiological and pathological processes in vascular cells^3^,^4^,^5^,^6^,^7^. It has been clearly demonstrated that ischemia-induced mitochondrial damage is a major risk factor for vascular endothelial cell apoptosis^8^,^9^,^10^, which can further lead to vascular diseases including vascular dementia, arteriosclerosis and brain oedema.

Since mitochondria are central to various kinds of cell death via the release of pro-apoptotic proteins from mitochondrial intermembrane space, including cytochrome c and Smac/DIABLO^11^,^12^, a key way to protect against vascular damage is through maintaining mitochondrial homeostasis. Recent evidences^13^,^14^,^15^,^16^ suggest that Bcl-2 protein family members are potent regulators of the mitochondrial changes during apoptosis, and two general drug intervention strategies of preventing mitochondrial depolarisation are practicable. The first is to enhance expression of mitochondria-related anti-apoptosis proteins, such as Bcl-2 and Bcl_XL_. The second approach is to downregulate mitochondria-related pro-apoptosis proteins like Bax, Bad, Bid and Bim. Especially in recent years, the question of how to promote Bcl-2 protein expression in dysfunctional mitochondria has become a major issue in the treatment of ischemia-injured vascular cells.

MicroRNAs (miRNAs) are a class of widely expressed endogenous short single strand non-coding RNA molecules which function in RNA silencing and post-transcriptional regulation of gene expression. Accumulating evidences show^17^,^18^,^19^ that miRNAs inhibit gene expression at the post-transcriptional level and exert key effects in cell proliferation, differentiation and apoptosis. Recently, it has been reported that some miRNAs such as miR-29, miR-30, miR-34, miR-125, miR-136, miR-181, miR-195 and miR-497 are involved in regulation of Bcl-2 protein function by inhibiting mRNA expression of the gene encoding Bcl-2, *BCL2*, under ischemic conditions^20^,^21^,^22^,^23^,^24^,^25^,^26^. Thus, these miRNAs constitute potential drug targets for vascular protection by specifically regulating Bcl-2 expression and maintaining mitochondrial homeostasis against ischemia insult.

In the present study, we found that the natural product 2,4,5-trihydroxybenzaldehyde (TDB) effectively protected vascular cells against oxygen-glucose deprivation/reperfusion (OGD/R)-induced insult by preventing the collapse of mitochondrial membrane potential (MMP) and thereby inhibiting activation of the caspase-dependent apoptosis pathway. We propose that TDB exerts this protective effect by specifically targeting miR-34a to increase *BCL2* mRNA expression. Taken together, our findings suggest that miR-34a is a promising novel drug target for the clinical treatment of ischemic vascular damage such as stroke, vascular dementia and brain oedema.

## Results

### TDB increased cell viability in OGD/R-induced vascular endothelial injury models

**Figure 1:**
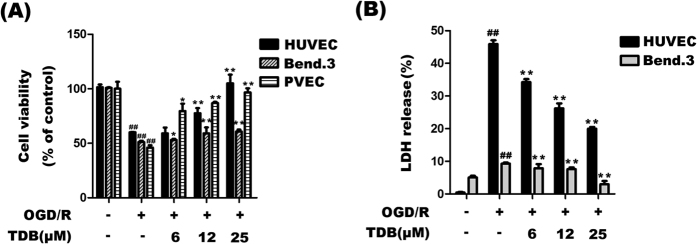
TDB protects from oxygen-glucose deprivation/reperfusion (OGD/R)-induced cell death. (**A**) HUVEC, Bend.3 and PVEC cells were subjected to oxygen-glucose deprivation (4 h) in the presence of TDB (6, 12 and 25 μM), then cultured in normoxic conditions for 24 h in the continued presence of TDB. Cell viability was assessed by MTT assay and expressed relative to untreated control cells. (**B**) HUVEC and Bend.3 cells were subjected to OGD/R and treated with TDB (6, 12 and 25 μM) as in (**A**). LDH release was expressed as a percentage of the maximum LDH release. All data are presented as mean ± S.D. from independent experiments performed in triplicate and statistical comparisons between the different groups were performed using one-way ANOVA with Tukey’s multiple comparison post-test. ^##^*P* < 0.01, relative to control group; *P < 0.05, **P < 0.01, relative to OGD group.

We first investigated the effect of TDB on OGD/R-induced injury in two different immortal vascular endothelial cell lines. OGD/R insult markedly decreased the cell viability of primary vascular endothelial cells (PVEC), HUVEC and Bend.3 cells measured using the MTT assay^27^, and this effect was dose-dependently reversed by TDB (6, 12, 25 μM; Fig. 1A). These results were confirmed using LDH leakage as a biomarker for cell toxicity. OGD/R insult significantly accelerated LDH release from HUVEC and Bend.3 cells, and the increase was dose-dependently reversed by TDB treatment (Fig. 1B). These results suggest that TDB maintains cell integrity and increases cell viability in OGD/R-induced vascular endothelial injury models.

### TDB protected vascular endothelial cells against OGD/R-induced apoptosis via caspase-9/3 pathway

**Figure 2:**
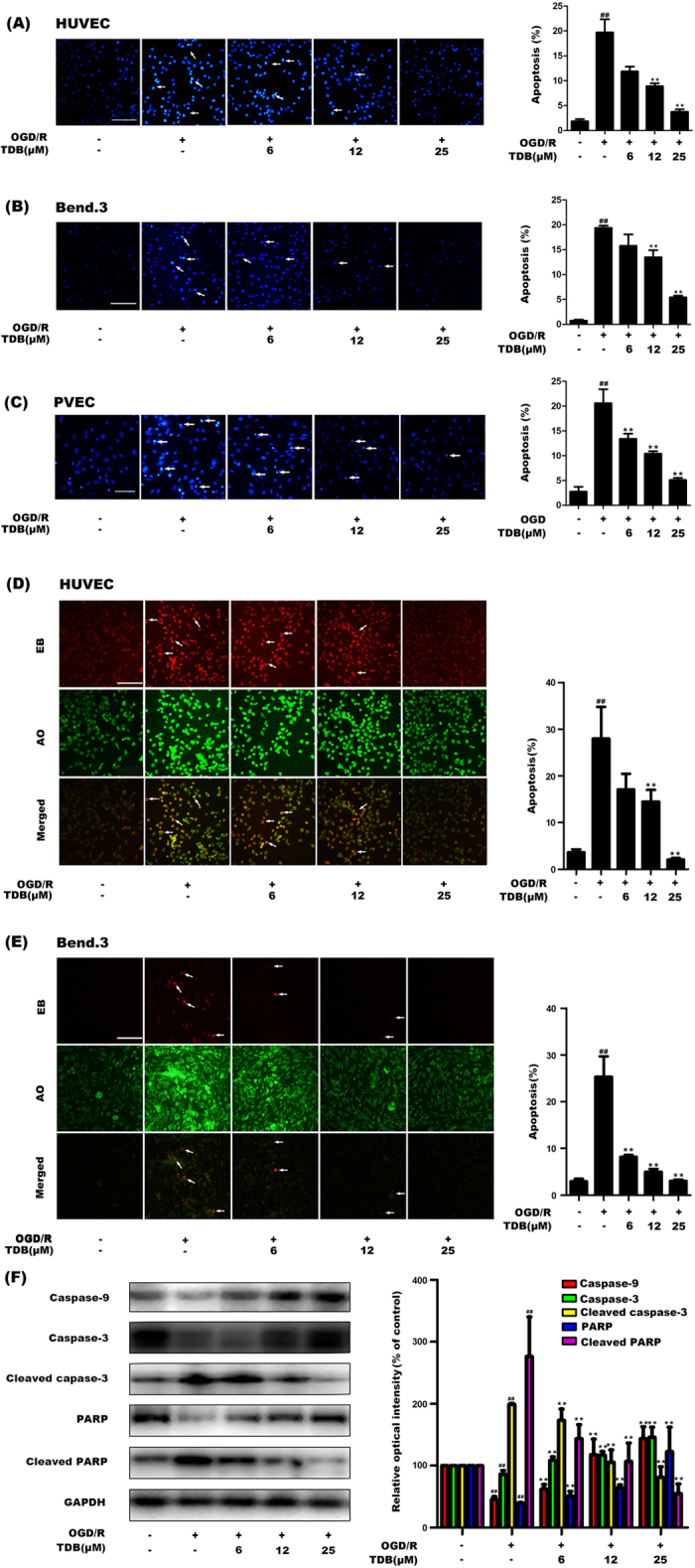
TDB protects vascular endothelial cells against OGD/R-mediated apoptosis via caspase-9/3 pathway. (**A**–**C**) HUVEC (**A**), Bend.3 (**B**) and PVEC (**C**) cells were subjected to oxygen-glucose deprivation (4 h) in the presence of TDB (6, 12 and 25 μM), then cultured in normoxic conditions for 24 h in the continued presence of TDB. Aptoptotic nuclei were identified using Hoechst 33258 staining (scale bar = 100 μm). (**D**,**E**) HUVEC (**D**) and Bend.3 (**E**) cells were subjected to oxygen-glucose deprivation (4 h) in the presence of TDB (6, 12 and 25 μM), then cultured in normoxic conditions for 24 h in the continued presence of TDB. Apoptotic cells were identified by double staining with acridine orange (AO) and ethidium bromide (EB). Cells which took up both dyes were classified as apoptotic (indicated by arrows in the EB and merged panels). Scale bar = 100 μm. (**F**) HUVEC cells were subjected to OGD/R and treated with TDB (6, 12 and 25 μM) as before, then lysed and immunoblotted for caspase-9, caspase-3, cleaved caspase-3, PARP and cleaved PARP. Data are presented as mean ± S.D. from independent experiments performed in triplicate and statistical comparisons between the different groups were performed using one-way ANOVA with Tukey’s multiple comparison post-test. ^##^*P* < 0.01, relative to control group; **P < 0.01, relative to OGD group.

To determine the effect of TDB specifically on apoptotic cell death, we used the DNA stain Hoechst 33258 as a sensitive assay for apoptosis. The nuclei of healthy cells stained by Hoechst 33258 showed uniform blue fluorescence, while apoptotic cells showed hyper-chromatic and dense fluorescent particles within the massive apoptotic nuclei or cytoplasm. Percentage apoptosis was calculated according to the number of apoptotic cells/the total number of cells × 100%. Our data revealed that OGD/R insult dramatically increased the proportion of apoptotic cells and that TDB treatment significantly protected against OGD/R-induced apoptosis in HUVEC cells (Fig. 2A), Bend.3 cells (Fig. 2B) and PVEC (Fig. 2C), These results were further supported by acridine orange/ethidium bromide (AO/EB) double staining analysis. AO enters both living and apoptotic cells and emits green fluorescence whereas EB only enters apoptotic cells and emits red fluorescence^28^. The AO/EB assay showed that the percentage of apoptotic cells significantly increased following OGD/R insult and decreased upon TDB treatment in both HUVEC (Fig. 2D) and Bend.3 (Fig. 2E) cells, suggesting that TDB could effectively inhibit the apoptosis of vascular endothelial cells induced by OGD/R insult. We next investigated the effects of TDB on the caspase-9/3 pathway, an important effector pathway for mitochondria-related apoptosis^29^,^30^. Immunoblotting revealed that OGD/R insult significantly increased the expressions of active (cleaved) forms of caspase-3 and PARP, and decreased the expressions of total caspase-9, caspase-3 and PARP in HUVEC cells and this effect was markedly reversed by TDB treatment (Fig. 2F), suggesting that TDB effectively protected vascular endothelial cells against apoptosis via regulation of mitochondria-dependent caspase-9/3 signalling pathway.

### TDB protected mitochondrial membrane integrity in OGD/R-induced vascular endothelial injury models

**Figure 3:**
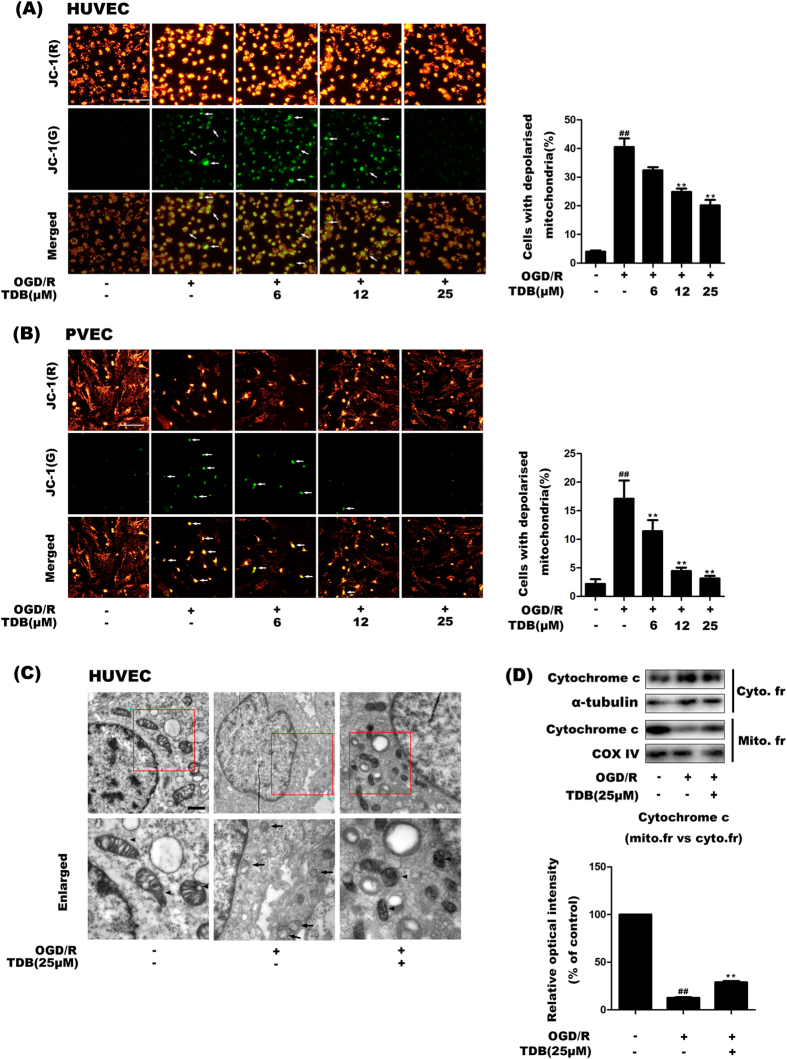
TDB protects mitochondrial membrane integrity. (**A**,**B**) JC-1 staining was used to investigate mitochondrial depolarisation in HUVEC cells (**A**) and PVEC (**B**) subjected to OGD/R injury with or without TDB treatment (6, 12 and 25 μM). Cells with depolarised mitochondria were identified by green fluorescence (example cells are indicated by arrows) and quantified as the percentage of the total cell number. Scale bar = 100 μm. (**C**) HUVEC cells were subjected to OGD/R injury and treated with TDB (25 μM) as before, then harvested and fixed for transmission electron microscopy. Images show the presence of abnormal mitochondria following OGD/R insult. Scale bar = 10 μm. (**D**) HUVEC cells were subjected to OGD/R injury and treated with TDB (25 μM) as in (**B**), then mitochondrial and cytosolic fractions were separated and cytochrome c expression was detected by western blot. Alpha-tubulin and COX IV were used as loading controls for the cytosolic and mitochondrial fractions respectively. All data are presented as mean ± S.D. from independent experiments performed in triplicate and statistical comparisons between the different groups were performed using one-way ANOVA with Tukey’s multiple comparison post-test. ^##^*P* < 0.01, relative to control group; *P < 0.05, **P < 0.01, relative to OGD group.

Mitochondria-related apoptosis is preceded by collapse of the mitochondrial membrane potential, rearrangements in mitochondrial ultrastructure, and the release of apoptotic signalling proteins such as cytochrome c from the intermembrane space. We assayed mitochondrial membrane potential in HUVEC cells under conditions of OGD/R using the potential-sensitive fluorophore JC-1. In living cells, JC-1 accumulates in mitochondria due to their negative charge and emits red fluorescence, while in cells with depolarised mitochondria, JC-1 exists as a monomer and emits green fluorescence^31^,^32^. The ratio of green fluorescence to red fluorescence can therefore reflect mitochondrial depolarisation. In our study, the number of cells with depolarised mitochondria (green fluorescence) was significantly increased following OGD/R and this increase was reversed by TDB treatment in a dose-dependent manner in HUVEC and PVEC (Fig. 3A and B). Next the ultrastructure of HUVEC cells was observed by transmission electron microscopy (TEM), which allowed morphologically normal and abnormal mitochondria to be clearly distinguished^33^,^34^. The TEM assay showed that the number of damaged mitochondria increased under OGD/R condition and this increase was again reversed by TDB treatment (Fig. 3C). Furthermore, mitochondrial and cytoplasmic distributions of cytochrome c, a key player in activation of caspase-dependent apoptosis pathway, were detected by western blot assay. We found that OGD/R insult caused a dramatic translocation of cytochrome c from mitochondria to cytoplasm which was significantly reduced by TDB treatment (Fig. 3D). These results suggested that TDB protects against mitochondrial depolarisation and subsequent mitochondria-related apoptosis in HUVEC cells.

### TDB upregulated pro-survival Bcl-2 protein expression by inhibiting *BCL2* mRNA degradation in oxygen-glucose deprivation-induced vascular endothelial injury models

**Figure 4:**
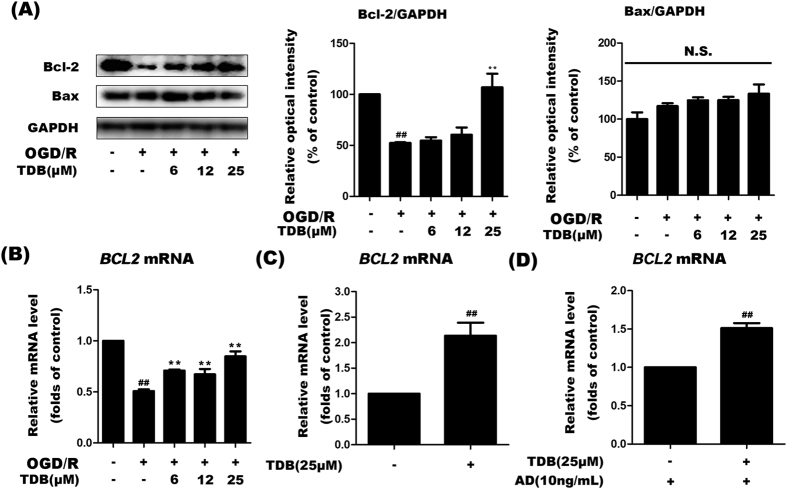
TDB upregulates pro-survival Bcl-2 protein expression by inhibiting *BCL2* mRNA degradation. (**A**) HUVEC cells were subjected to ODG/R injury and treated with TDB (6, 12 and 25 μM) as previously, then protein levels of Bcl-2 and Bax were assessed by immunoblotting and normalised to GADPH protein levels. (**B**) The effect of ODG/R insult and TDB treatment on *BCL2* mRNA levels was assessed by RT-PCR. (**C**) The effect of TDB (25 μM) treatment for 24 h under normoxic conditions on *BCL2* mRNA expression was assessed by RT-PCR. (**D**) The effect of TDB treatment (25 μM) on *BCL2* mRNA expression was assessed in the presence of the transcriptional inhibitor actinomycin D (AD; 10 ng/ml). All protein and mRNA expression data were normalised to expression levels in untreated control cells and presented as mean ± S.D. from independent experiments performed in triplicate. Statistical comparisons between the different groups were performed using one-way ANOVA with Tukey’s multiple comparison post-test. ^##^*P* < 0.01, relative to control group; *P < 0.05, **P < 0.01, relative to OGD group.

Bcl-2 is an anti-apoptotic protein which has been shown to maintain mitochondrial membrane integrity^35^. Western blotting revealed that Bcl-2 protein expression decreased upon OGD insult and this effect was dose-dependently reversed by TDB treatment, suggesting that Bcl-2 may be a crucial target protein of TDB in the regulation of mitochondria-related apoptosis pathway (Fig. 4A). RT-PCR analysis showed that OGD/R insult also dramatically decreased expression of Bcl-2 at the mRNA level, which was dose-dependently increased by TDB treatment (Fig. 4B). Furthermore, we observed that TDB still induced an increase in *BCL2* mRNA expression under normoxic conditions (Fig. 4C). This increase in *BCL2* mRNA expression could not be blocked by actinomycin D (AD), a transcription inhibitor^36^ (Fig. 4D), suggesting that TDB treatment promotes an increase in *BCL2* mRNA levels by supressing its degradation rather than by increasing its transcription.

### TDB decreased miR34a expression to stabilise *BCL2* mRNA in oxygen-glucose deprivation-induced vascular endothelial injury models

**Figure 5:**
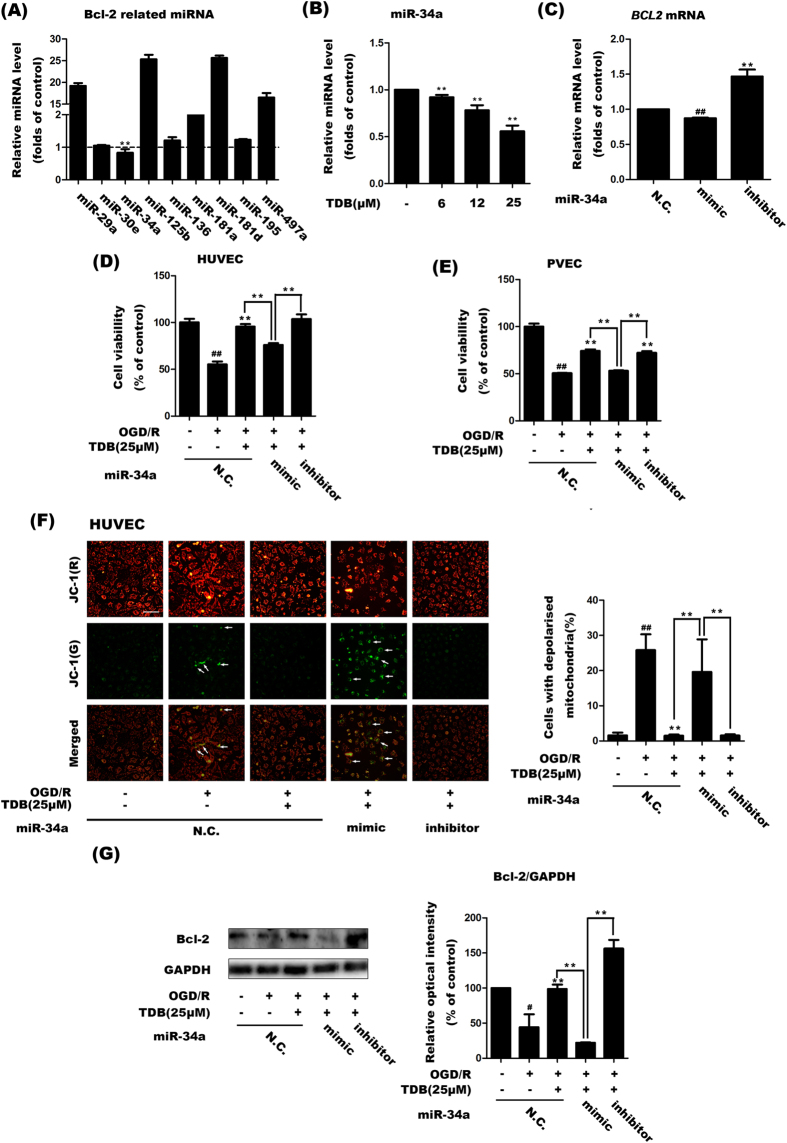
TDB decreases miR34a expression to stabilise *BCL2* mRNA. (**A**) The effect of TDB treatment (25 μM for 24 h) on levels of Bcl-2-related miRNAs was assessed by RT-PCR. (**B**) The dose-dependence of TDB-mediated suppression of miR-34a expression was assessed by RT-PCR. HUVEC cells were treated with TDB (6, 12 and 25 μM) for 24 h under normoxic conditions. (**C**) HUVEC cells transfected with negative control miRNA (N.C.; 100 ng/ml), miR-34a mimic (100 ng/ml) or miR-34a inhibitor (100 ng/ml) were harvested and *BCL2* mRNA levels were assessed by RT-PCR. (**D**–**G**) HUVEC cells or PVEC were transfected with negative control miRNA (N.C.; 100 ng/ml), miR-34a mimic (100 ng/ml) or miR-34a inhibitor (100 ng/ml), then 24 h later were subjected to OGD/R injury and treatment with TDB (25 μM) as previously described. Cells were then assayed for viability by MTT assay (**D**, **E**), for mitochondrial depolarisation using JC-1 (**F**), and for Bcl-2 protein levels by western blot (**G**). All data are presented as mean ± S.D. from independent experiments performed in triplicate and statistical comparisons between the different groups were performed using one-way ANOVA with Tukey’s multiple comparison post-test. ^#^*P* < 0.05, ^##^*P* < 0.01, relative to control group; *P < 0.05, **P < 0.01, relative to OGD group.

It has been confirmed that the TDB-induced up-regulation of Bcl-2 resulted from the inhibition of Bcl-2 mRNA degradation, which is closely related to microRNA. MicroRNAs (miRNAs) are a class of widely expressed endogenous short single strand non-coding RNA molecules which function in RNA silencing and post-transcriptional regulation of gene expression, suggesting that RNA stabilisation was involved by miRNA. Therefore, to further explore the mechanism of TDB-dependent *BCL2* mRNA stabilisation, we measured the expression of miRNAs which have been reported to specifically target *BCL2* mRNA and promote its degradation^37^. First, we analysed the expression of a series of Bcl-2-related miRNAs in TDB-treated HUVEC cells. The result showed that miR-34a expression significantly decreased following TDB treatment (25 μM), while expression of other miRNAs (including miR-30e, miR-115b, miR-125b and others) increased or did not change (Fig. 5A). Further study confirmed that miR-34a expression was dose-dependently downregulated by TDB treatment (Fig. 5B). We therefore speculated that TDB might inhibit miR-34a expression and thereby promote *BCL2* mRNA stabilisation. To further investigate this hypothesis, we used an inhibitor or a mimic^38^ of miR-34a to investigate the relationship between miR-34a expression and the protective effect of TDB on vascular endothelial cells. As expected, the miR-34a mimic significantly decreased *BCL2* mRNA expression while the inhibitor significantly increased *BCL2* mRNA (Fig. 5C). Interestingly, the miR-34a mimic markedly blocked the protective effect of TDB on cell viability in HUVEC cells (Fig. 5D) and PVEC (Fig. 5E) subjected to OGD/R, supporting the hypothesis that TDB protects from OGD/R by downregulating miR-34a expression. Moreover, staining with JC-1 revealed that while OGD/R-induced mitochondrial depolarisation was decreased under TDB treatment, it was significantly increased upon co-treatment of TDB and the miR-34a mimic (Fig. 5F). Western blotting revealed that Bcl-2 protein expression decreased following OGD/R injury and that the TDB-induced increase of Bcl-2 protein expression was markedly reversed by the miR-34a mimic (Fig. 5G). By contrast, transfection with the miR-34a inhibitor produced the opposite effect on cell viability, mitochondrial membrane potential, *BCL2* mRNA and protein expression (Fig. 5D–G). Taken together, these results suggest that TDB decreases miR-34a expression to stabilise *BCL2* mRNA, resulting in upregulation of Bcl-2 protein and consequent protection against OGD/R-induced apoptosis in vascular endothelial cells.

### TDB protects vascular endothelial cells *in vivo*

**Figure 6:**
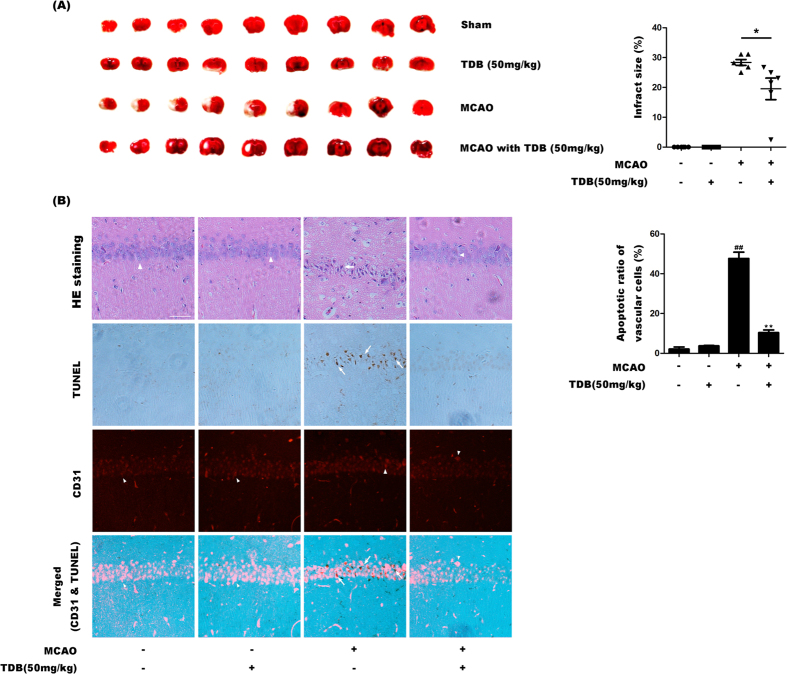
TDB protects vascular endothelial cells *in vivo*. (**A**) SD rats were subjected to MCAO (1 h) with the pre-treatment of TDB (50 mg/kg). Then, the coronal brain sections were cut and stained with 1% TTC. The red regions indicated viable brain tissue, whereas non-stained pale regions indicated infarct brain tissue (n = 6). (**B**) SD rats were subjected to MCAO (1 h) with the pre-treatment of TDB (50 mg/kg). Then, the coronal brain sections were cut and stained with HE, TUNEL or the antibody of CD31 in fluorescence IHC assay (Scale bar = 50 μm, n = 6, arrows indicate apoptotic vascular cells, arrow heads indicate normal vascular cells). Data are presented as means ± S.D. and statistical comparisons between the different groups were performed using one-way ANOVA with Tukey’s multiple comparison post-test. ^##^*P* < 0.01, relative to sham group; *P < 0.05, **P < 0.01, relative to MCAO group.

The results *in vitro* proved that TBD was a promising lead compound for the treatment of ischemic cerebral vascular diseases. To confirm our findings, more experiments *in vivo* are necessary. It is well known that middle cerebral artery occlusion (MCAO) results in injury to the striatum, cortex, and hippocampus region^39^,^40^,^41^, which can be clearly visualized by TTC staining. TTC which is usually white in color is reduced by dehydogenases (especially succinate dehydrogenase) into pink formazon in the viable tissue, and the injured or infarcted brain region remains white in color^42^. The result revealed that the MCAO group showed large and readily detectable infarct confined to striatum and cortex region along with some areas of hippocampus region (Fig. 6A). However, the infarct was markedly reduced in TDB treated groups compared with MCAO group. HE staining showed that ischemia induced obvious cerebral neuronal structure changes, such as vacuolation and karyopyknosis, in the hippocampus CA1 areas. However, these pathological changes were effectively reversed in TDB-treated group, suggesting a marked protective effect of TDB on cerebral vascular cells under ischemia insult (Fig. 6B). Moreover, the apoptotic ratio of cerebrovascular cells was calculated in red fluorescence stained with CD31 and brown in TUNEL assay. CD31 was a marker of vascular cells^43^. Thus, vascular cells were marked in red fluorescence and counted. Moreover, TUNEL assay was used to detect apoptotic cells that undergo extensive DNA degradation during the late stages of apoptosis. The assay principle is based on the presence of nicks in the damaged DNA which can be recognized by terminal deoxynucleotidyl transferase or TdT^44^. The apoptotic cells were stained in brown in TUNEL assay. Apoptotic vascular cells was identified and counted, for the cells emitted red fluorescence in IHC assay with CD31 and stained in brown in TUNEL assay. The apoptotic ratio of vascular cells was calculated as: Number of apoptotic vascular cells/Number of total vascular cells × 100%. The results showed that MCAO insult markedly increased the apoptotic ratio of cerebrovascular cells and TDB significant reversed the increase induced by MCAO. These *in vivo* findings suggested that TBD was a promising lead compound for the treatment of ischemic cerebral vascular diseases.

## Discussion

Mitochondrial outer membrane integrity is mediated primarily by a group of evolutionarily conserved proteins known as the Bcl-2 protein family. Recent research shows that Bcl-2 protein, a key member of the Bcl-2 family, plays a central role in the survival of vascular endothelial cells by maintaining mitochondrial membrane potential and suppressing mitochondria-dependent apoptosis under ischemic conditions. Multiple studies^13^,^15^,^45^ have suggested that downregulation of mitochondrial Bcl-2 destabilises the integrity of the mitochondrial outer membrane and enhances the release of cytochrome c from mitochondria, leading to activation of the caspase-mediated apoptosis pathway. In the present study, we observed that TDB markedly protected vascular endothelial cells against ischemic damage through increasing mitochondrial Bcl-2 protein expression and maintaining mitochondrial membrane potential. This protective effect of TDB was independent of the glycolytic metabolism by vascular cells, for TDB attenuated cell death induced by OGD/R insult in both high and normal glucose medium (data not shown). Further mechanistic investigation revealed that TDB negatively regulates the expression of miR-34a, which could specifically target and degrade *BCL2* mRNA sequence, resulting in the stabilisation of *BCL2* mRNA.

Previous studies^13^,^14^,^15^,^16^ have revealed that activation of anti-apoptotic Bcl-2 protein or inactivation of pro-apoptotic Bax protein could disturb the process of vascular endothelial cell apoptosis under ischemic conditions. In this study, we found that TDB significantly increased Bcl-2 protein expression in a dose-dependent manner without affecting Bax expression. Interestingly, this finding was somewhat different from a previous report on the effect of protocatechuic aldehyde, a chemical structurally similar to TDB, on the regulation of Bcl-2 and Bax. Since protocatechuic aldehyde did not show any effect on Bcl-2 or Bax expression^46^, and the only difference between the two structures is that TDB possesses an additional phenolic hydroxyl group compared to protocatechuic aldehyde, we speculate that this phenolic hydroxyl group might be responsible for TDB-dependent Bcl-2 protein expression regulation. Of course, this will need further investigation in the future.

Since TDB significantly upregulated *BCL2* mRNA levels in a dose-dependent manner, we speculated that the TDB-dependent increase in Bcl-2 protein might result from either activation of *BCL2* gene expression or stabilisation of *BCL2* mRNA. It should be noted that TDB could significantly increase *BCL2* mRNA levels compared with the control group (untreated cells) even when gene transcription was blocked by actinomycin D (AD). This finding indicates that TDB stabilises existing *BCL2* mRNA rather than increasing its transcription.

Several strategies may be proposed to target Bcl-2, including suppression of *BCL2* gene transcription, prevention of Bcl-2 protein translation, or inhibition of Bcl-2 protein function by direct interaction or by promoting its degradation. Among these approaches, an attractive possibility for achieving functional regulation of Bcl-2 protein is to activate or inhibit specific miRNAs that target the *BCL2* mRNA sequence. Several previous studies have revealed that *BCL2* is a target of several miRNAs, such as miR-30e which downregulates *BCL2* gene expression to promote apoptosis through the cleavage of caspases^18^, and miR-34a which inhibits *BCL2* gene expression to induce sensitivity to the anti-tumour effect of sorafenib in human HCC cells^20^. Furthermore, miR-29a, miR-125b, miR-136, miR-181, miR-195 and miR-497 modulate Bcl-2 expression to induce apoptosis of neurons and breast cancer cells by targeting *BCL2* mRNA^22^,^23^,^24^,^25^,^26^. In particular, a recent report indicated that miR-34a negatively regulated Bcl-2 protein expression at the post-transcriptional level and induced mitochondrial damage. This effect could result in the collapse of mitochondria and induce cell apoptosis. In the present study, our findings suggest that miR-34a is the major target of TDB and is markedly downregulated by TDB, causing a dramatic stabilisation of *BCL2* mRNA and protein levels. Continuous and stable expression of Bcl-2 further promoted the maintenance of mitochondrial membrane potential and cell survival under ischemia condition (Fig. 6).

In support of our hypothesis, we found that a miR-34a mimic could significantly reverse the protective effect of TDB on mitochondrial membrane potential and vascular endothelial cell viability under ischemia conditions, while a miR-34a inhibitor showed the opposite effect. Moreover, previous *in vitro* research suggested that miR-34a could damage the blood-brain barrier in cerebrovascular endothelial cell monolayer by downregulating mitochondrial oxidative phosphorylation and suppressing the production of ATP^47^. Therefore, we speculate that TDB might be used as a cerebrovascular protective candidate for treatment of vascular dementia and brain oedema in clinical trials.

**Figure 7:**
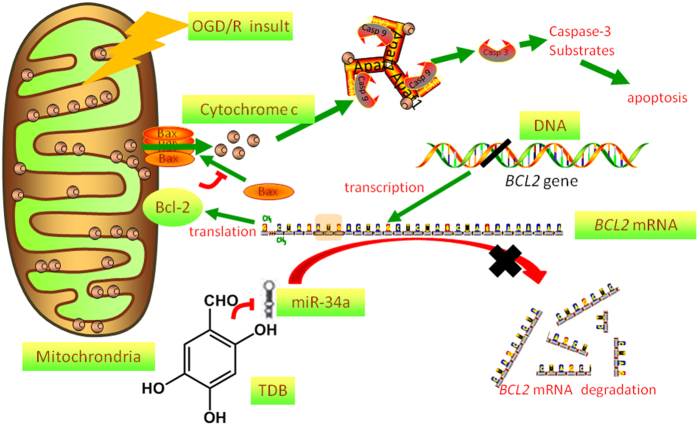
Proposed mechanism of TDB-mediated protection against OGD insult in vascular endothelial cells. TDB suppresses expression of the miRNA miR-34a, preventing its degradation of *BCL2* mRNA and thus increasing Bcl-2 protein levels. Bcl-2 maintains mitochondrial membrane potential and opposes mitochondrial outer membrane permeabilisation mediated by Bax, preventing release of cytochrome c from the intermembrane space and consequent activation of caspases 3 and 9.

In this study, we investigated the protective effect of TDB on vascular endothelial cells against OGD/R insult and found that TDB exerts its protective effect by targeting miR-34a. TDB suppressed miR-34a expression to stabilise *BCL2* mRNA, which resulted in upregulation of Bcl-2 protein expression and maintenance of mitochondria membrane integrity (Fig. 7). These results suggest that miR-34a could be a novel drug target for protecting vascular endothelial cells against ischemic injury.

## Methods

### Materials

2,4,5-trihydroxybenzaldehyde (TDB, C_7_H_6_O_4_) was purchased from Tokyo Chemistry Industry (Tokyo, Japan). The molecular weight of TDB was 122. High-performance liquid chromatography showed the purity of TDB was 98%. 3-[4,5-dimethylthiazol-2-yl] 2,5-diphenyltetrazolium bromide (MTT) and Hoechst 33258 were purchased from Sigma Chemical Co. (St Louis, MO, USA). LDH assay kit, JC-1 kit and TUNEL apoptosis assay kit were from Beyotime Institute of Biotechnology (Nanjing, Jiangsu, China). Protein A/G-agarose was from Biogot Technology Co. (Nanjing, Jiangsu, China). Fetal bovine serum (FBS), High glucose Dulbecco’s Modified Eagle medium (DMEM, 4.5 g/L glucose), antibiotics, and trypsin were from Hyclone (Logan, UT, USA). Endothelial Cell Medium (ECM) was from ScienCell (Carlsbad, CA, USA). Anaero Pack and Anaero container were perchased from Mitsubishi GAS CHEMICAL (Minato-ku, Tokyo, Japan). The primary antibodies and HRP conjugated goat anti-rabbit IgG were purchased from Cell Signaling Technology (Beverly, MA, USA). Chemiluminescent HRP substrate was purchased from Pierce Scientific (IL, USA).

### Cell culture

HUVEC cells were obtained from Peking Union Medical College Cell Bank (Beijing, China) and grown in high-glucose DMEM medium supplemented with 10% FBS, 100 U/ml penicillin, and 100 μg/ml streptomycin at 37 °C in a humidified incubator with 95% air and 5% CO_2_. Bend.3 cells were obtained from Peking Union Medical College, Cell Bank (Beijing, China) and grown in Endothelial Cell Medium with 2% FBS, 100 U/ml penicillin, and 100 μg/ml streptomycin at 37 °C in a humidified incubator with 95% air and 5% CO_2_. Primary vascular endothelial cells (PVEC) were isolated from embryonic (E18 days) Kunming mouse fetuses (Vital River, Beijing, China). The isolation was performed in PBS on the ice. Briefly, the whole lung was firstly isolated from the embryo. Then, the lungs were cut into 1 mm^3^ fragments and the fragments were moved into a T175 culture flask. Finally, the fragments were scattered and DMEM medium supplemented with 10% FBS was added into the flask. Then, the primary vascular endothelial cells (PVEC) were cultured for 7 days.

### Oxygen-glucose deprivation/reperfusion (OGD/R) insult

Before the experiment, HUVEC or Bend.3 cells were cultured in the medium as described above. Then, the medium was replaced with Earle’s balanced salt solution (Leagene Biotech Co. Beijing, China), and the cells were placed into the sealed Anaero container with an Anaero Pack (Mitsubishi, Tokyo, Japan) for 4 h to initiate the OGD insult. OGD was then terminated by adding fresh complete medium and the cells were cultured for an additional 24 h under normoxic condition at 37 °C.

### TDB treatment

HUVEC or Bend.3 cells were treated with TDB (6, 12 and 25 μM) under OGD conditions for 4 h. OGD was then terminated and the cells were cultured for a further 20 h under normoxic conditions in the presence of TDB.

### MTT Assay

HUVEC and Bend.3 cells were cultured and subjected to OGD/R insult and TDB treatment as described previously. After 24 h under normoxic conditions, culture supernatants were replaced by MTT solution (5 mg/ml) and incubated for 4 h at 37 °C. The supernatant was then removed and 100 μl of dimethyl sulfoxide (DMSO) was added. The optical density (OD) was measured at 570 nm. Cell viability (%) = [OD (OGD/R treatment) − OD (blank)]/[OD (control) − OD (blank)] × 100%.

### Lactate dehydrogenase (LDH) assay

HUVEC and Bend.3 cells were cultured and subjected to OGD/R insult and TDB treatment as described previously. Then, the LDH released from cells was detected using a commercial LDH kit according to the manufacturer’s instructions. Absorbance was measured at 450 nm and the relative LDH release rate was calculated as follows: LDH release rate (%) = [OD (treatment) − OD (blank)]/[OD (Max.) − OD (blank)] × 100%.

### Hoechst 33258 staining and AO/EB staining

For Hoechst 33258 staining, HUVEC or Bend.3 cells were subjected to OGD/R insult and TDB treatment as described previously. The cells were then fixed in 4% paraformaldehyde and incubated with Hoechst 33258 working solution (1 μg/ml) for 30 min at room temperature. Images were captured using a fluorescence microscope (IX73, Olympus, Japan) under excitation wavelength 352 nm and emission wavelength 461 nm. For AO/EB staining, no fixation was carried out. The cells were washed with PBS and treated with the AO/EB mixture (2 μg/ml) for 2 min at room temperature, whereupon the cells were again washed three times with PBS. The cells were visualised using a fluorescence microscope under excitation wavelength 490 nm and emission wavelength 530 nm for AO staining and excitation wavelength 520 nm and emission wavelength 590 nm for EB staining.

### Mitochondrial membrane potential assay

HUVEC cells were subjected to OGD/R insult and TDB treatment as described previously. Mitochondrial depolarisation was then assessed using a commercial JC-1 assay kit. Briefly, the cells were incubated with JC-1 solution (10 μg/ml) at 37 °C for 20 min in dark, then were washed three times with PBS and visualised using a fluorescence microscope under excitation wavelength 460 nm and emission wavelength 530 nm for JC-1 monomer, and excitation wavelength 520 nm and emission wavelength 590 nm for JC-1 polymer. Cells were considered to have depolarised mitochondria if they exhibited green (monomeric) fluorescence; cells exhibiting red (polymeric) fluorescence were considered to have normal mitochondria.

### Transmission electron microscope (TEM) assay

Cells were harvested and fixed in 4% pre-chilled glutaraldehyde overnight at 4 °C. The cells were then washed four times with PBS, followed by post-fixation with 1% OsO_4_ in 0.1 M PBS for 2 h at 4 °C. Samples were dehydrated in graded ethanol, embedded in Epon 812, and subsequently cut into ultra- or semi-thin sections. The sections were examined under a transmission electron microscope (Hitachi HT7700-SS; Hitachi, Tokyo, Japan).

### Western blot assay

After treatment as above, the cells were harvested and lysed in RIPA buffer (1X) with cocktail protein inhibitors. The lysates were separated by 8–15% SDS-PAGE and electrotransferred to nitrocellulose membranes. For the detection of cytochrome c, mitochondria were separated from the cytosolic fraction using the Mitochondria-Cytosol isolation kit (Beyotime Institute of Biotechnology, China). The membranes were blocked in 5% non-fat milk and then incubated with primary antibody (1:1000) at room temperature for 2 h and further incubated with secondary antibody (1:1000) for 1 h. Then, the membranes were washed and visualised by enhanced chemiluminescent substrate and scanned with the Kodak Digital Imaging System. The bands were visualised using Carestream Molecular Imaging Software (Carestream Health, Inc., Rochester, NY, USA).

### Real-time polymerase chain reaction (RT-PCR)

Total RNA was extracted using a cell/bacterial total mRNA Purification Kit (TIANGEN, Beijing, China). Total RNA was reverse transcribed at 42 °C for 50 min using a RevertAid First Strand cDNA Synthesis Kit (TIANGEN, Beijing, China) to obtain cDNA. Then, cDNA was amplified using TransStarts Green qPCR SuperMix (Transgen, Beijing, China). *GAPDH* was used as the internal control. Real-time PCR was carried out on the Agilent Technologies Stratagene Mx3005 P (USA). The program for PCR reactions was 94 °C for 10 min followed by 40 cycles of 94 °C for 30 s, 56 °C for 30 s and 72 °C for 30 s. The *BCL2* and *GAPDH* primer pairs for real-time PCR are shown in Table 1. At the end of real-time PCR, the CT values of each reaction were determined and the changes in transcriptional level of *BCL2* gene normalised to *GAPDH* were calculated relative to mRNA level of *BCL2* gene in untreated cells (folds of control).

To measure miRNA levels, total miRNA was extracted using a miRcute miRNA Purification Kit (TIANGEN, Beijing, China). Total miRNA was reverse transcribed at 37 °C for 60 min using a miRcute miRNA First-Strand cDNA Synthesis Kit (TIANGEN, Beijing, China) to obtain cDNA. cDNA was then amplified using miRcute miRNA qPCR Detection Kit (SYBR Green)(TIANGEN, Beijing, China). U6 was taken as the internal control. Real-time PCR was carried out on the Agilent Technologies Stratagene Mx3005 P (USA). The program for PCR reactions was 94 °C for 2 min followed by 40 cycles of 94 °C for 20 s, 60 °C for 34 s. The forward primers for miRNAs and U6 are shown in Table 2. At the end of real-time PCR, the CT values of each reaction were provided and the changes in transcriptional level of miRNAs normalized to U6 were calculated.

### miRNA transfection

The cells were first transfected with negative control miRNA, miR-34a mimic or miR-34a inhibitor (GenePharma, Shanghai, China) for 24 h using Lipofectamine RNAiMAX (Invitrogen, CA, USA) according to the manufacturer’s introductions. The cells were then treated with TDB (25 μM) under OGD condition for 4 h, whereupon OGD was terminated and the cells were further cultured for 24 h under normoxic conditions. The cells were then assayed by MTT assay, MMP assay and western blot.

### Animals

The experimental protocol was approved by the Ethical Institutional Animal Care and Use Committee of Peking University (EIACUC-PKU), and all animal experiments were performed in accordance with the Ethical Guidelines of EIACUC-PKU. Male SD rats weighting 250 g–280 g were purchased from VITAL RIVER Laboratories (Beijing, China). Rats were kept in animal house at 25 ± 1 °C under constant dark and light cycles. Animals were provided with water and standard pellet diet freely throughout the experiment. The SD rats were divided into four groups. Group 1 was sham group; group 2 was sham group with TDB treatment (50 mg/kg); group 3 was MCAO model group, and group 4 was MCAO model group with TDB treatment (50 mg/kg). The animals in group 4 were pre-treated with TDB for 1 h, and then MCAO models were established. After 24 h of MCAO, the rats were sacrificed and the brains were disserted out immediately for ischemic region analysis by TTC staining or vascular pathological analysis by hematoxylin and eosin (HE) staining, immunohistochemistry with CD31 and TUNEL.

### Cerebral ischemia *in vivo*

The experimental protocol was approved by the Ethical Institutional Animal Care and Use Committee of Peking University. Right MCAO was performed by an intraluminal filament model^39^. Ischemia was induced for 1 h and the suture was withdrawn slowly. However, in sham group, external carotid artery (ECA) was only exposed but suture was not inserted actually. Afterward, animals were kept in their cages for 24 h for reperfusion.

### Infarct analysis

SD rats were sacrificed and brains were collected and placed in brain matrix after removing the hind region. 3 mm coronal brain sections were cut and stained with 0.1% TTC solution^44^. TTC was reduced by succinate dehydrogenase (mitochondria) into red formazan in the viable region, and it remained unstained or colourless in the nonviable infarct region.

### HE staining

The rats were anaesthetized, sacrificed, and their brains were dissected out immediately, post fixed, and embedded with paraffin. Coronal sections with thicknesses of 5 μm with cortex region were dewaxed and stained with hematoxylin and eosin, and studied under microscope.

### Fluorescence immunohistochemistry (IHC) assay with CD31

Immunohistochemistry was done according to the method described by Feng *et al*.^48^ with some modifications to detect the expressions of CD31 proteins.

### TUNEL assay

The terminal deoxynucleotidyl transferase mediated dUTP-biotin nick end labeling (TUNEL) assay was used to observe apoptotic vascular cells, following the protocols of the former paper described^49^. Brown stained dots were indications of apoptotic cells. The apoptotic vascular cells were identified and counted.

### Statistical analysis

All experiments were performed at least three times. Statistical data are expressed as means ± standard deviation (S.D.) Comparisons among different groups were carried out with Student’s t-test and analysis of variance (ANOVA). Values of *P* < 0.05 were considered to be statistically significant.

## Additional Information

**How to cite this article**: Liao, L.-X. *et al*. TDB protects vascular endothelial cells against oxygen-glucose deprivation/reperfusion-induced injury by targeting miR-34a to increase Bcl-2 expression. *Sci. Rep*. **6**, 37959; doi: 10.1038/srep37959 (2016).

**Publisher’s note:** Springer Nature remains neutral with regard to jurisdictional claims in published maps and institutional affiliations.

## Figures and Tables

**Table 1:** Primer pairs for real-time PCR.

Gene	Sequence
*BCL2*	**F:** 5′-CGCATCAGGAAGGCTAGAGTT-3′
	**R:** 5′-CAGACATTCGGAGACCACACT-3′
*GAPDH*	**F:** 5′-AGAAGGCTGGGGCTCATTTG-3′
	**R:** 5′-AGGGGCCATCCACAGTCTTC-3′

**Table 2:** Forward primers of miRNAs for real-time PCR.

miRNA	Sequence
miR-29	5′-GCUGGUUUCAUAUGGUGGUUUAGA-3′
miR-30e	5′-CUUUCAGUCGGAUGUUUACAGC-3′
miR-125b	5′-UCCCUGAGACCCUAACUUGUGA-3′
miR-136	5′-ACUCCAUUUGUUUUGAUGAUGGA-3′
miR-181a	5′-AACAUUCAACGCUGUCGGUGAGU-3′
miR-181d	5′-AACAUUCAUUGUUGUCGGUGGGU-3′
miR-195	5′-UAGCAGCACAGAAAUAUUGGC-3′
miR-497a	5′-CAGCAGCACACUGUGGUUUGU-3′
U6	5′-GGGCAGGAAGAGGGCCTAT-3′
